# Supplementary material for: Differential Effects of Human Tau Isoforms to Neuronal Dysfunction and Toxicity in the Drosophila CNS
Source: Int J Mol Sci. 2022 Oct 26;23(21):12985. doi: 10.3390/ijms232112985 (PMC9655709; doi:10.3390/ijms232112985)
Supplement: Supplementary file 1 [file ijms-23-12985-s001.zip › ijms-1951703-supplementary.pdf]

International Journal of *Molecular Sciences*

Supplementary

Differential Effects of Human Tau Isoforms to Neuronal Dysfunction and Toxicity in the Drosophila CNS

Ergina Vourkou [1,2,†], Vassilis Paspaliaris [1,3,†], Anna Bourouliti [1,4,], Maria-Christina Zerva [1,5], Engie Prifti [1,6], Katerina Papanikolopoulou [1,*] and Efthimios M. C. Skoulakis [1,*]

1 Institute for Fundamental Biomedical Research, Biomedical Sciences Research Centre "Alexander Fleming", 16672 Vari, Greece
2 School of Medicine, National and Kapodistrian University of Athens, 11527 Athens, 2nd Department of Neurology, "Attikon" General University Hospital, 12462 Athens, Greece
3 Laboratory of Experimental Physiology, School of Medicine, National and Kapodistrian University of Athens, 11527 Athens, Greece
4 Department of Molecular Biology and Genetics, Democritus University of Thrace, 68100 Alexandroupolis, Greece
5 Athens International Master's Program in Neurosciences, Department of Biology, National and Kapodistrian University of Athens, 15784 Athens, Greece
6 Department of Biotechnology, Agricultural University of Athens, 11855 Athens, Greece
* Correspondence: papanikolopoulou@fleming.gr (K.P.); skoulakis@fleming.gr (E.M.C.S.)
† These authors contributed equally to this work.

Citation: Vourkou, E.; Paspaliaris, V.; Bourouliti, A.; Zerva, M.-C.; Prifti, E.; Papanikolopoulou, K.; Skoulakis, E.M.C Differential Effects of Human Tau Isoforms to Neuronal Dysfunction and Toxicity in the Drosophila CNS. *Int. J. Mol. Sci.* **2022**, *23*, 12985. https://doi.org/10.3390/ijms232112985

Academic Editor(s): Isidro Ferrer

Received: 19 September 2022
Accepted: 19 October 2022
Published: date

Publisher's Note: MDPI stays neutral with regard to jurisdictional claims in published maps and institutional affiliations.

Copyright: © 2022 by the authors. Licensee MDPI, Basel, Switzerland. This article is an open access article distributed under the terms and conditions of the Creative Commons Attribution (CC BY) license (https://creativecommons.org/licenses/by/4.0/).

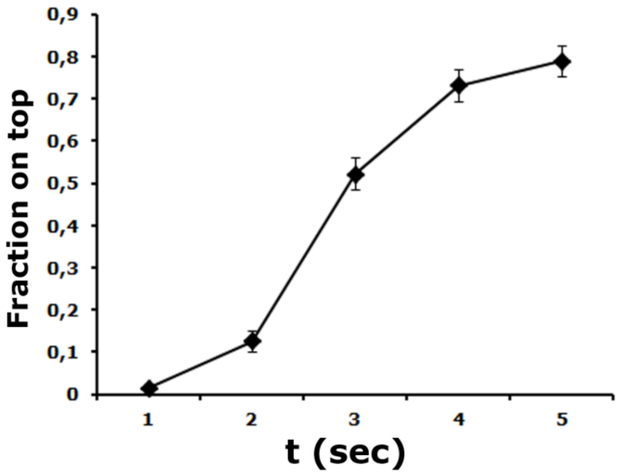

Supplemental Figure S1. A "standard curve" of top upper compartment occupation.

The proportion of flies reported as a mean± SEM in the top upper compartment as a function of time after forcing a population of 10-12 flies to the bottom of the vial. n=19

Supplemental Table S1.

ANOVAs and subsequent LSM-planned comparisons for the ratios of densitometrically determined protein **(Fig.1A)** or mRNA **(Fig.1B)** levels of each of the six hTau isoforms normalized for loading with Syntaxin **(A)** or *rp49* **(B).** Significant differences are highlighted in bold.

Statistical details from Figure 1			
**Genotype**	**Mean ± SEM**	**F-Ratio**	**p**
**Figure 1A**	**ANOVA $F_{(5,17)}$=5.7256, p=0.0063**		
$Elav^{C155}$-Gal4 >0N3R	0.5079 ± 0.0992		
$Elav^{C155}$-Gal4 >1N3R	0.9109 ± 0.1852	4.4104	0.0575
$Elav^{C155}$-Gal4 >2N3R	0.4788 ± 0.0966	0.0230	0.8818
$Elav^{C155}$-Gal4 >0N4R	0.9860 ± 0.2017	6.2080	**0.0283**
$Elav^{C155}$-Gal4 >1N4R	1.2739 ± 0.0930	15.936	**0.0018**
$Elav^{C155}$-Gal4 >2N4R	0.5375 ± 0.0875	0.0238	0.8799
$Elav^{C155}$-Gal4 >1N3R	0.9109 ± 0.1852		
$Elav^{C155}$-Gal4 >2N3R	0.4788 ± 0.0966	5.0711	**0.0438**
$Elav^{C155}$-Gal4 >0N4R	0.9860 ± 0.2017	0.1533	0.7023
$Elav^{C155}$-Gal4 >1N4R	1.2739 ± 0.0930	3.5793	0.0829
$Elav^{C155}$-Gal4 >2N4R	0.5375 ± 0.0875	3.7857	0.0755
$Elav^{C155}$-Gal4 >2N3R	0.4788 ± 0.0966		
$Elav^{C155}$-Gal4 >0N4R	0.9860 ± 0.2017	6.9876	**0.0214**
$Elav^{C155}$-Gal4 >1N4R	1.2739 ± 0.0930	17.171	**0.0014**
$Elav^{C155}$-Gal4 >2N4R	0.5375 ± 0.0875	0.0938	0.7647
$Elav^{C155}$-Gal4 >0N4R	0.9860 ± 0.2017		
$Elav^{C155}$-Gal4 >1N4R	1.2739 ± 0.0930	2.2512	0.1593
$Elav^{C155}$-Gal4 >2N4R	0.5375 ± 0.0875	5.4624	**0.0376**
$Elav^{C155}$-Gal4 >1N4R	1.2739 ± 0.0930		
$Elav^{C155}$-Gal4 >2N4R	0.5375 ± 0.0875	14.727	**0.0024**
**Genotype**	**Mean ± SEM**	**F-Ratio**	**p**
**Figure 1B**	**ANOVA $F_{(5,23)}$=0.9896, p=0.4513**		
$Elav^{C155}$-Gal4 >0N3R	0.5772 ± 0.0834		
$Elav^{C155}$-Gal4 >1N3R	0.5461 ± 0.0794	0.0403	0.8431
$Elav^{C155}$-Gal4 >2N3R	0.5433 ± 0.1246	0.0479	0.8291
$Elav^{C155}$-Gal4 >0N4R	0.6229 ± 0.1751	0.0874	0.7709
$Elav^{C155}$-Gal4 >1N4R	0.6378 ± 0.0771	0.1532	0.7001
$Elav^{C155}$-Gal4 >2N4R	0.3364 ± 0.0802	2.4220	0.1370
$Elav^{C155}$-Gal4 >1N3R	0.5461 ± 0.0794		
$Elav^{C155}$-Gal4 >2N3R	0.5433 ± 0.1246	0.0003	0.9856
$Elav^{C155}$-Gal4 >0N4R	0.6229 ± 0.1751	0.2464	0.6256

Elav^{C155}-Gal4 >1N4R	0.6378 ± 0.0771	0.3506	0.5611
Elav^{C155}-Gal4 >2N4R	0.3364 ± 0.0802	1.8375	0.1920
Elav^{C155}-Gal4 >2N3R	0.5433 ± 0.1246		
Elav^{C155}-Gal4 >0N4R	0.6229 ± 0.1751	0.2648	0.6131
Elav^{C155}-Gal4 >1N4R	0.6378 ± 0.0771	0.3725	0.5492
Elav^{C155}-Gal4 >2N4R	0.3364 ± 0.0802	1.7884	0.1978
Elav^{C155}-Gal4 >0N4R	0.6229 ± 0.1751		
Elav^{C155}-Gal4 >1N4R	0.6378 ± 0.0771	0.0092	0.9247
Elav^{C155}-Gal4 >2N4R	0.3364 ± 0.0802	3.4295	0.0805
Elav^{C155}-Gal4 >1N4R	0.6378 ± 0.0771		
Elav^{C155}-Gal4 >2N4R	0.3364 ± 0.0802	3.7934	0.0672

Supplemental Table S2.

Figure 2A. The means and SEMs of the quantification of the area of the mushroom body calyces of the indicated genotypes are shown. Following the indicated significant ANOVA the means were compared using Dunnett's tests with the means of the driver heterozygotes

Figure 2B. The means and SEMs for the learning performance of the indicated genotypes are shown. ANOVAs and subsequent planned multiple comparisons of the indicated genotypes are shown. Significant differences are highlighted in bold.

**Statistical details from Figure 2**			
**Genotype**	**Mean ± SEM**	**Dunnetts' p**	
**Figure 2A**	**ANOVA $F_{(6,63)}$=3.3319, p=0.0070**		
Elav^{C155}-Gal4> w^{1118}	0.1737 ± 0.0073	1	
Elav^{C155}-Gal4 >0N3R	0.1520 ± 0.0053	**0.0311**	
Elav^{C155}-Gal4 >1N3R	0.1439 ± 0.0057	**0.0096**	
Elav^{C155}-Gal4 >2N3R	0.1459 ± 0.0099	**0.0363**	
Elav^{C155}-Gal4 >0N4R	0.1431 ± 0.0049	**0.0028**	
Elav^{C155}-Gal4 >1N4R	0.1631 ± 0.0039	0.2349	
Elav^{C155}-Gal4 >2N4R	0.1620 ± 0.0049	0.2135	
**Genotype**	**Mean ± SEM**	**F-Ratio**	**p**
**Figure 2B- 3 pairings for 3R isoforms**	**ANOVA $F_{(6,93)}$= 2.2081, p= 0.0497**		
w^{1118}>0N3R	60.100 ± 2.411		
Elav^{C155}-Gal4> w^{1118}	61.228 ± 2.108	0.0855	0.7707
Elav^{C155}-Gal4 >0N3R	50.348 ± 3.175	6.1597	**0.0150**
Elav^{C155}-Gal4> w^{1118}	61.228 ± 2.108		
Elav^{C155}-Gal4 >0N3R	50.348 ± 3.175	7.9512	**0.0059**

w^{1118}>1N3R	59.257 ± 2.495		
$Elav^{C155}$-Gal4> w^{1118}	61.228 ± 2.108	0.2609	0.6108
$Elav^{C155}$-Gal4 >1N3R	56.463 ± 2.230	0.5244	0.4709
$Elav^{C155}$-Gal4> w^{1118}	61.228 ± 2.108		
$Elav^{C155}$-Gal4 >1N3R	56.463 ± 2.230	1.5837	0.2116
w^{1118}>2N3R	53.512 ± 2.819		
$Elav^{C155}$-Gal4> w^{1118}	61.228 ± 2.108	3.9987	0.0486
$Elav^{C155}$-Gal4 >2N3R	60.442 ± 3.575	3.2254	0.0760
$Elav^{C155}$-Gal4> w^{1118}	61.228 ± 2.108		
$Elav^{C155}$-Gal4 >2N3R	60.442 ± 3.575	0.0431	0.8360
$Elav^{C155}$-Gal4> 0N3R	50.348 ± 3.175		
$Elav^{C155}$-Gal4 >1N3R	56.463 ± 2.230	2.5118	0.1166
$Elav^{C155}$-Gal4> 2N3R	60.442 ± 3.575	6.8438	**0.0105**
$Elav^{C155}$-Gal4 >1N3R	56.463 ± 2.230		
$Elav^{C155}$-Gal4> 2N3R	60.442 ± 3.575	1.1043	0.2962
**Figure 2B- 3 pairings for 4R isoforms ANOVA $F_{(6,84)}$=21.033, p=1.62e-14**			
w^{1118}>0N4R	73.072 ± 1.445		
$Elav^{C155}$-Gal4> w^{1118}	63.466 ± 2.583	7.3483	0.0082
$Elav^{C155}$-Gal4 >0N4R	46.521 ± 2.820	51.628	**3.49e-10**
$Elav^{C155}$-Gal4> w^{1118}	63.466 ± 2.583		
$Elav^{C155}$-Gal4 >0N4R	46.521 ± 2.820	21.830	**1.22e-5**
w^{1118}>1N4R	59.458 ± 3.095		
$Elav^{C155}$-Gal4> w^{1118}	63.466 ± 2.583	1.2790	0.2615
$Elav^{C155}$-Gal4 >1N4R	37.582 ± 3.384	35.048	**8.16e-8**
$Elav^{C155}$-Gal4> w^{1118}	63.466 ± 2.583		
$Elav^{C155}$-Gal4 >1N4R	37.582 ± 3.384	50.941	**4.31e-10**
w^{1118}>2N4R	57.781 ± 2.147		
$Elav^{C155}$-Gal4> w^{1118}	63.466 ± 2.583	2.6806	0.1056
$Elav^{C155}$-Gal4 >2N4R	48.241 ± 2.085	7.5490	**0.0074**
$Elav^{C155}$-Gal4> w^{1118}	63.466 ± 2.583		
$Elav^{C155}$-Gal4 >2N4R	48.241 ± 2.085	19.226	**3.58e-5**
$Elav^{C155}$-Gal4> 0N4R	46.521 ± 2.820		
$Elav^{C155}$-Gal4 >1N4R	37.582 ± 3.384	5.6088	**0.0203**
$Elav^{C155}$-Gal4> 2N4R	48.241 ± 2.085	0.2248	0.6367

Elav^{C155}-Gal4 >1N4R	37.582 ± 3.384		
Elav^{C155}-Gal4> 2N4R	48.241 ± 2.085	8.6386	**0.0043**
**Figure 2B- 6 pairings for 3R isoforms**	**ANOVA $F_{(6,81)}$=0.3956, p=0.8796**		
w^{1118}>0N3R	80.295 ± 2.842		
Elav^{C155}-Gal4> w^{1118}	77.078 ± 2.026	0.9899	0.3229
Elav^{C155}-Gal4 >0N3R	79.239 ± 2.740	0.1027	0.7495
Elav^{C155}-Gal4> w^{1118}	77.078 ± 2.026		
Elav^{C155}-Gal4 >0N3R	79.239 ± 2.740	0.4678	0.4961
w^{1118}>1N3R	81.389 ± 2.029		
Elav^{C155}-Gal4> w^{1118}	77.078 ± 2.026	1.7783	0.1864
Elav^{C155}-Gal4 >1N3R	79.841 ± 2.656	0.2117	0.6467
Elav^{C155}-Gal4> w^{1118}	77.078 ± 2.026		
Elav^{C155}-Gal4 >1N3R	79.841 ± 2.656	0.7304	0.3955
w^{1118}>2N3R	79.882 ± 2.334		
Elav^{C155}-Gal4> w^{1118}	77.078 ± 2.026	0.7877	0.3776
Elav^{C155}-Gal4 >2N3R	81.000 ± 1.240	0.1205	0.7294
Elav^{C155}-Gal4> w^{1118}	77.078 ± 2.026		
Elav^{C155}-Gal4 >2N3R	81.000 ± 1.240	1.5414	0.2183
Elav^{C155}-Gal4> 0N3R	79.239 ± 2.740		
Elav^{C155}-Gal4 >1N3R	79.841 ± 2.656	0.0334	0.8554
Elav^{C155}-Gal4> 2N3R	81.000 ± 1.240	0.2989	0.5862
Elav^{C155}-Gal4 >1N3R	79.841 ± 2.656		
Elav^{C155}-Gal4> 2N3R	81.000 ± 1.240	0.1238	0.7259
**Figure 2B- 6 pairings for 4R isoforms**	**ANOVA $F_{(6,93)}$=61.567, p=3.26e-29**		
w^{1118}>0N4R	79.571 ± 2.269		
Elav^{C155}-Gal4> w^{1118}	73.833 ± 3.074	2.7719	0.0995
Elav^{C155}-Gal4 >0N4R	38.217 ± 3.145	138.65	**1.07e-19**
Elav^{C155}-Gal4> w^{1118}	73.833 ± 3.074		
Elav^{C155}-Gal4 >0N4R	38.217 ± 3.145	102.84	**2.08e-16**
w^{1118}>1N4R	72.427 ± 2.147		
Elav^{C155}-Gal4> w^{1118}	73.833 ± 3.074	0.1537	0.6960
Elav^{C155}-Gal4 >1N4R	35.622 ± 1.630	105.27	**1.19e-16**
Elav^{C155}-Gal4> w^{1118}	73.833 ± 3.074		
Elav^{C155}-Gal4 >1N4R	35.622 ± 1.630	122.93	**2.53e-18**
w^{1118}>2N4R	71.816 ± 1.570		

Elav^{C155}-Gal4> w^{1118}	73.833 ± 3.074	0.3426	0.5598
Elav^{C155}-Gal4 >2N4R	41.500 ± 3.093	74.508	**2.55e-13**
Elav^{C155}-Gal4> w^{1118}	73.833 ± 3.074		
Elav^{C155}-Gal4 >2N4R	41.500 ± 3.093	84.754	**1.70e-14**
Elav^{C155}-Gal4> 0N4R	38.217 ± 3.145		
Elav^{C155}-Gal4 >1N4R	35.622 ± 1.630	0.5458	0.4620
Elav^{C155}-Gal4> 2N4R	41.500 ± 3.093	0.8430	0.3611
Elav^{C155}-Gal4 >1N4R	35.622 ± 1.630		
Elav^{C155}-Gal4> 2N4R	41.500 ± 3.093	2.8015	0.0978

Supplemental Table S3.

The means and SEMs for PSD-M **(A)** and PSI-M **(B)** performance of the indicated genotypes are shown. Following the indicated ANOVA, the means were compared using planned multiple comparisons. Significant differences are highlighted in bold.

**Statistical details from Figure 3**				
	**Genotype**	**Mean ± SEM**	**F-Ratio**	**p**
**Figure 3A**		**ANOVA $F_{(2,31)}$=2.039, p=0.1484**		
**PSD-M 0N3R**	w^{1118}>0N3R	48.416 ± 5.244		
	Elav^{C155}-Gal4> w^{1118}	38.933 ± 2.948	2.8796	0.1004
	Elav^{C155}-Gal4 >0N3R	38.238 ± 3.454	3.3173	0.0789
	Elav^{C155}-Gal4> w^{1118}	38.933 ± 2.948		
	Elav^{C155}-Gal4 >0N3R	38.238 ± 3.454	0.0162	0.8994
**Figure 3A**		**ANOVA $F_{(2,31)}$=0.9166, p=0.4111**		
**PSD-M 1N3R**	w^{1118}>1N3R	32.001 ± 3.085		
	Elav^{C155}-Gal4> w^{1118}	29.293 ± 3.997	0.2708	0.6067
	Elav^{C155}-Gal4 >1N3R	35.797 ± 3.309	0.5526	0.4632
	Elav^{C155}-Gal4> w^{1118}	29.293 ± 3.997		
	Elav^{C155}-Gal4 >1N3R	35.797 ± 3.309	1.8106	0.1889
**Figure 3A**		**ANOVA $F_{(2,31)}$= 2.6441, p=0.0881**		
**PSD-M 2N3R**	w^{1118}>2N3R	43.165 ± 5.819		
	Elav^{C155}-Gal4> w^{1118}	33.281 ± 3.195	2.6209	0.1163
	Elav^{C155}-Gal4 >2N3R	46.364 ± 3.300	0.2996	0.5883
	Elav^{C155}-Gal4> w^{1118}	33.281 ± 3.195		
	Elav^{C155}-Gal4 >2N3R	46.364 ± 3.300	5.0097	0.033

**Figure 3A**		**ANOVA $F_{(2,31)}$=25.692, p=3.78e-7**		
**PSD-M 0N4R**	w^{1118}>0N4R	49.098 ± 3.427		
	$Elav^{C155}$-Gal4> w^{1118}	44.864 ± 2.658	1.1858	0.2851
	$Elav^{C155}$-Gal4 >0N4R	23.415 ± 2.049	43.636	**3.07e-7**
	$Elav^{C155}$-Gal4> w^{1118}	44.864 ± 2.658		
	$Elav^{C155}$-Gal4 >0N4R	23.415 ± 2.049	31.957	**4.14e-6**
**Figure 3A**		**ANOVA $F_{(2,23)}$=10.429, p=0.0007**		
**PSD-M 1N4R**	w^{1118}>1N4R	39.195 ± 5.235		
	$Elav^{C155}$-Gal4> w^{1118}	43.778 ± 3.199	0.856	0.3654
	$Elav^{C155}$-Gal4 >1N4R	23.589 ± 1.667	10.466	**0.0040**
	$Elav^{C155}$-Gal4> w^{1118}	43.778 ± 3.199		
	$Elav^{C155}$-Gal4 >1N4R	23.589 ± 1.667	18.840	**0.0003**
**Figure 3A**		**ANOVA $F_{(2,31)}$= 5.324, p=0.011**		
**PSD-M 2N4R**	w^{1118}>2N4R	58.560 ± 2.084		
	$Elav^{C155}$-Gal4> w^{1118}	57.202 ± 3.983	0.0610	0.8066
	$Elav^{C155}$-Gal4 >2N4R	43.277 ± 4.406	8.4326	**0.0070**
	$Elav^{C155}$-Gal4> w^{1118}	57.202 ± 3.983		
	$Elav^{C155}$-Gal4 >2N4R	43.277 ± 4.406	7.0004	**0.0130**
**Figure 3B**		**ANOVA $F_{(2,28)}$=0.2831, p=0.7557**		
**PSI-M 0N3R**	w^{1118}>0N3R	33.850 ± 3.560		
	$Elav^{C155}$-Gal4> w^{1118}	32.922 ± 1.972	0.0489	0.8267
	$Elav^{C155}$-Gal4 >0N3R	30.678 ± 3.326	0.5416	0.4683
	$Elav^{C155}$-Gal4> w^{1118}	32.922 ± 1.972		
	$Elav^{C155}$-Gal4 >0N3R	30.678 ± 3.326	0.2711	0.6070
**Figure 3B**		**ANOVA $F_{(2,32)}$= 0.1413, p=0.8688**		
**PSI-M 1N3R**	w^{1118}>1N3R	23.380 ± 3.583		
	$Elav^{C155}$-Gal4> w^{1118}	24.400 ± 2.140	0.0415	0.8400
	$Elav^{C155}$-Gal4 >1N3R	25.904 ± 4.011	0.2780	0.6019
	$Elav^{C155}$-Gal4> w^{1118}	24.400 ± 2.140		
	$Elav^{C155}$-Gal4 >1N3R	25.904 ± 4.011	0.0937	0.7616
**Figure 3B**		**ANOVA $F_{(2,34)}$=0.2812, p=0.7567**		
**PSI-M 2N3R**	w^{1118}>2N3R	28.552 ± 2.957		
	$Elav^{C155}$-Gal4> w^{1118}	31.151 ± 2.779	0.3062	0.5839
	$Elav^{C155}$-Gal4 >2N3R	31.972 ± 4.330	0.5071	0.4816
	$Elav^{C155}$-Gal4> w^{1118}	31.151 ± 2.779		
	$Elav^{C155}$-Gal4 >2N3R	31.972 ± 4.330	0.0292	0.8654

**Figure 3B**		**ANOVA $F_{(2,39)}$=2.9498, p=0.0648**		
**PSI-M 0N4R**	w^{1118}>0N4R	35.173 ± 2.745		
	$Elav^{C155}$-Gal4> w^{1118}	43.757 ± 2.150	5.7798	0.0213
	$Elav^{C155}$-Gal4 >0N4R	40.708 ± 2.730	2.3168	0.1365
	$Elav^{C155}$-Gal4> w^{1118}	43.757 ± 2.150		
	$Elav^{C155}$-Gal4 >0N4R	40.708 ± 2.730	0.7294	0.3986
**Figure 3B**		**ANOVA $F_{(2,33)}$=0.6895, p=0.5094**		
**PSI-M 1N4R**	w^{1118}>1N4R	31.781 ± 1.846		
	$Elav^{C155}$-Gal4> w^{1118}	27.737 ± 4.595	0.6890	0.4128
	$Elav^{C155}$-Gal4 >1N4R	26.394 ± 2.803	1.3192	0.2595
	$Elav^{C155}$-Gal4> w^{1118}	27.737 ± 4.595		
	$Elav^{C155}$-Gal4 >1N4R	26.394 ± 2.803	0.0864	0.7707
**Figure 3B**		**ANOVA $F_{(2,38)}$=0.6591, p=0.5235**		
**PSI-M 2N4R**	w^{1118}>2N4R	30.034 ± 2.343		
	$Elav^{C155}$-Gal4> w^{1118}	33.859 ± 2.202	1.0727	0.3072
	$Elav^{C155}$-Gal4 >2N4R	33.379 ± 3.135	0.8559	0.3610
	$Elav^{C155}$-Gal4> w^{1118}	33.859 ± 2.202		
	$Elav^{C155}$-Gal4 >2N4R	33.379 ± 3.135	0.0163	0.8991

Supplemental Table S4.

Figure 4A. The means and SEMs for odor and electric footshock avoidance performance of the indicated genotypes are shown. Following the indicated ANOVA, the means were compared using planned multiple comparisons.

Figure 4B. ANOVAs followed by planned multiple comparisons using the LSM approach of the indicated genotypes in each compartment of the vial are shown. Significant differences are highlighted in bold.

**Statistical details from Figure 4**				
	**Genotype**	**Mean ± SEM**	**F-Ratio**	**p**
**Figure 4A-Avoidance BNZ**				
		**ANOVA $F_{(2,27)}$=3.8713, p=0.0343**		
**0N3R**	w^{1118}>0N3R	67.668 ± 2.805		
	$Elav^{C155}$-Gal4> w^{1118}	56.070 ± 3.383	7.1742	0.0129
	$Elav^{C155}$-Gal4 >0N3R	60.329 ± 2.595	3.6543	0.0674
	$Elav^{C155}$-Gal4> w^{1118}	56.070 ± 3.383		
	$Elav^{C155}$-Gal4 >0N3R	60.329 ± 2.595	1.0051	0.3257

	**ANOVA $F_{(2,31)}$=3.2074, p=0.0552**			
**1N3R**	w^{1118}>1N3R	72.462 ± 3.519		
	$Elav^{C155}$-Gal4> w^{1118}	64.410 ± 1.618	4.3100	0.0468
	$Elav^{C155}$-Gal4 >1N3R	63.834 ± 2.501	5.1954	**0.0302**
	$Elav^{C155}$-Gal4> w^{1118}	64.410 ± 1.618		
	$Elav^{C155}$-Gal4 >1N3R	63.834 ± 2.501	0.0220	0.8831
	**ANOVA $F_{(2,31)}$= 1.0711, p=0.3558**			
**2N3R**	w^{1118}>2N3R	69.918 ± 2.771		
	$Elav^{C155}$-Gal4> w^{1118}	69.989 ± 3.852	0.0002	0.9886
	$Elav^{C155}$-Gal4 >2N3R	63.981 ± 3.629	1.7520	0.1960
	$Elav^{C155}$-Gal4> w^{1118}	69.989 ± 3.852		
	$Elav^{C155}$-Gal4 >2N3R	63.981 ± 3.629	1.3947	0.2472
	**ANOVA $F_{(2,22)}$=2.9509, p=0.0753**			
**0N4R**	w^{1118}>0N4R	80.557 ± 2.977		
	$Elav^{C155}$-Gal4> w^{1118}	71.875 ± 4.043	4.1227	0.0558
	$Elav^{C155}$-Gal4 >0N4R	72.347 ± 1.832	4.3376	0.0503
	$Elav^{C155}$-Gal4> w^{1118}	71.875 ± 4.043		
	$Elav^{C155}$-Gal4 >0N4R	72.347 ± 1.832	0.0116	0.9154
	**ANOVA $F_{(2,21)}$=0.6821, p=0.5175**			
**1N4R**	w^{1118}>1N4R	73.833 ± 5.416		
	$Elav^{C155}$-Gal4> w^{1118}	69.610 ± 4.673	0.4936	0.4908
	$Elav^{C155}$-Gal4 >1N4R	66.817 ± 2.119	1.3623	0.2576
	$Elav^{C155}$-Gal4> w^{1118}	69.610 ± 4.673		
	$Elav^{C155}$-Gal4 >1N4R	66.817 ± 2.119	0.2518	0.6215
	**ANOVA $F_{(2,31)}$=2.2191, p=0.1268**			
**2N4R**	w^{1118}>2N4R	70.103 ± 2.433		
	$Elav^{C155}$-Gal4> w^{1118}	62.208 ± 3.267	3.6279	0.0668
	$Elav^{C155}$-Gal4 >2N4R	69.838 ± 3.293	0.0041	0.9494
	$Elav^{C155}$-Gal4> w^{1118}	62.208 ± 3.267		
	$Elav^{C155}$-Gal4 >2N4R	69.838 ± 3.293	3.1060	0.0885
**Figure 4A-Avoidance OCT**				
	**ANOVA $F_{(2,28)}$=1.0050, p=0.3798**			
**0N3R**	w^{1118}>0N3R	64.160 ± 2.106		
	$Elav^{C155}$-Gal4> w^{1118}	60.137 ± 2.463	1.6054	0.2164
	$Elav^{C155}$-Gal4 >0N3R	60.997 ± 1.854	1.2760	0.2689
	$Elav^{C155}$-Gal4> w^{1118}	60.137 ± 2.463		
	$Elav^{C155}$-Gal4 >0N3R	60.997 ± 1.854	0.0733	0.7887

	**ANOVA $F_{(2,31)}$=4.6176, p=0.0182**			
1N3R	w^{1118}>1N3R	69.539 ± 2.505		
	$Elav^{C155}$-Gal4> w^{1118}	60.338 ± 2.763	6.1407	0.0193
	$Elav^{C155}$-Gal4 >1N3R	59.594 ± 2.553	7.5331	**0.0103**
	$Elav^{C155}$-Gal4> w^{1118}	60.338 ± 2.763		
	$Elav^{C155}$-Gal4 >1N3R	59.594 ± 2.553	0.0402	0.8425
	**ANOVA $F_{(2,31)}$=1.7471, p=0.1921**			
2N3R	w^{1118}>2N3R	61.107 ± 3.008		
	$Elav^{C155}$-Gal4> w^{1118}	54.299 ± 3.781	2.1009	0.1579
	$Elav^{C155}$-Gal4 >2N3R	63.043 ± 2.610	0.2329	0.6330
	$Elav^{C155}$-Gal4> w^{1118}	54.299 ± 3.781		
	$Elav^{C155}$-Gal4 >2N3R	63.043 ± 2.610	3.3671	0.0768
	**ANOVA $F_{(2,21)}$=1.3749, p=0.2769**			
0N4R	w^{1118}>0N4R	65.477 ± 3.356		
	$Elav^{C155}$-Gal4> w^{1118}	68.808 ± 4.046	0.4830	0.4955
	$Elav^{C155}$-Gal4 >0N4R	60.983 ± 2.585	1.0259	0.3238
	$Elav^{C155}$-Gal4> w^{1118}	68.808 ± 4.046		
	$Elav^{C155}$-Gal4 >0N4R	60.983 ± 2.585	2.6657	0.1190
	**ANOVA $F_{(2,23)}$=2.1611, p=0.1401**			
1N4R	w^{1118}>1N4R	69.832 ± 2.775		
	$Elav^{C155}$-Gal4> w^{1118}	64.562 ± 2.692	1.6844	0.2084
	$Elav^{C155}$-Gal4 >1N4R	61.201 ± 3.042	4.2837	0.0510
	$Elav^{C155}$-Gal4> w^{1118}	64.562 ± 2.692		
	$Elav^{C155}$-Gal4 >1N4R	61.201 ± 3.042	0.7369	0.4004
	**ANOVA $F_{(2,34)}$=16.7994, p=1.0282e-5**			
2N4R	w^{1118}>2N4R	74.369 ± 1.835		
	$Elav^{C155}$-Gal4> w^{1118}	59.167 ± 2.333	25.934	1.518e-5
	$Elav^{C155}$-Gal4 >2N4R	62.103 ± 1.717	23.447	**3.141e-5**
	$Elav^{C155}$-Gal4> w^{1118}	59.167 ± 2.333		
	$Elav^{C155}$-Gal4 >2N4R	62.103 ± 1.717	1.0519	0.3127
**Figure 4A-Shock Avoidance**				
	**ANOVA $F_{(2,23)}$=0.7404, p=0.4890**			
0N3R	w^{1118}>0N3R	86.739 ± 1.974		
	$Elav^{C155}$-Gal4> w^{1118}	86.339 ± 1.923	0.0233	0.8802
	$Elav^{C155}$-Gal4 >0N3R	89.703 ± 1.848	1.0233	0.3232
	$Elav^{C155}$-Gal4> w^{1118}	86.339 ± 1.923		

	$Elav^{C155}$-Gal4 >0N3R	89.703 ± 1.848	1.3181	0.2638
	**ANOVA $F_{(2,40)}$=2.7349, p=0.0777**			
**1N3R**	w^{1118}>1N3R	90.892 ± 1.625		
	$Elav^{C155}$-Gal4> w^{1118}	94.775 ± 0.835	5.0948	0.0298
	$Elav^{C155}$-Gal4 >1N3R	91.861 ± 0.962	0.3294	0.5694
	$Elav^{C155}$-Gal4> w^{1118}	94.775 ± 0.835		
	$Elav^{C155}$-Gal4 >1N3R	91.861 ± 0.962	2.8696	0.0984
	**ANOVA $F_{(2,27)}$=0.9972, p=0.3831**			
**2N3R**	w^{1118}>2N3R	94.092 ± 0.938		
	$Elav^{C155}$-Gal4> w^{1118}	96.304 ± 0.902	1.9914	0.1705
	$Elav^{C155}$-Gal4 >2N3R	95.147 ± 1.283	0.5094	0.4820
	$Elav^{C155}$-Gal4> w^{1118}	96.304 ± 0.902		
	$Elav^{C155}$-Gal4 >2N3R	95.147 ± 1.283	0.5450	0.4672
	**ANOVA $F_{(2,22)}$=0.2171, p=0.8067**			
**0N4R**	w^{1118}>0N4R	87.784 ± 0.770		
	$Elav^{C155}$-Gal4> w^{1118}	86.339 ± 1.923	0.2119	0.6502
	$Elav^{C155}$-Gal4 >0N4R	88.466 ± 4.225	0.0381	0.8471
	$Elav^{C155}$-Gal4> w^{1118}	86.339 ± 1.923		
	$Elav^{C155}$-Gal4 >0N4R	88.466 ± 4.225	0.3901	0.5393
	**ANOVA $F_{(2,22)}$=0.3978, p=0.6770**			
**1N4R**	w^{1118}>1N4R	85.754 ± 1.829		
	$Elav^{C155}$-Gal4> w^{1118}	86.339 ± 1.923	0.0412	0.8412
	$Elav^{C155}$-Gal4 >1N4R	83.832 ± 2.426	0.3953	0.5366
	$Elav^{C155}$-Gal4> w^{1118}	86.339 ± 1.923		
	$Elav^{C155}$-Gal4 >1N4R	83.832 ± 2.426	0.7566	0.3947
	**ANOVA $F_{(2,23)}$=0.1596, p=0.8535**			
**2N4R**	w^{1118}>2N4R	87.766 ± 1.785		
	$Elav^{C155}$-Gal4> w^{1118}	86.339 ± 1.923	0.2126	0.6495
	$Elav^{C155}$-Gal4 >2N4R	87.945 ± 3.116	0.0029	0.9573
	$Elav^{C155}$-Gal4> w^{1118}	86.339 ± 1.923		
	$Elav^{C155}$-Gal4 >2N4R	87.945 ± 3.116	0.2502	0.6221
**Figure 4B**				
**bottom**	**ANOVA $F_{(6,97)}$=0.6687, p=0.6751**			
	$Elav^{C155}$-Gal4> w^{1118}	0.1775 ± 0.0257		
	$Elav^{C155}$-Gal4 >0N3R	0.1694 ± 0.0429	0.0264	0.8713
	$Elav^{C155}$-Gal4 >1N3R	0.2006 ± 0.0319	0.2414	0.6243
	$Elav^{C155}$-Gal4 >2N3R	0.2496 ± 0.0408	2.4371	0.1220

	$Elav^{C155}$-Gal4 >0N4R	0.2093 ± 0.0325	0.4578	0.5003
	$Elav^{C155}$-Gal4 >1N4R	0.2334 ± 0.0457	1.2859	0.2598
	$Elav^{C155}$-Gal4 >2N4R	0.2202 ± 0.0344	0.7522	0.3880
**middle**	**ANOVA $F_{(6,97)}$= 1.2078, p= 0.3095**			
	$Elav^{C155}$-Gal4> w^{1118}	0.3009 ± 0.0413		
	$Elav^{C155}$-Gal4 >0N3R	0.2966 ± 0.0410	0.0056	0.9405
	$Elav^{C155}$-Gal4 >1N3R	0.2925 ± 0.0460	0.0234	0.8786
	$Elav^{C155}$-Gal4 >2N3R	0.2581 ± 0.0298	0.6380	0.4265
	$Elav^{C155}$-Gal4 >0N4R	0.3759 ± 0.0331	1.8826	0.1734
	$Elav^{C155}$-Gal4 >1N4R	0.2715 ± 0.0529	0.2646	0.6082
	$Elav^{C155}$-Gal4 >2N4R	0.3731 ± 0.0432	1.5895	0.2106
**upper**	**ANOVA $F_{(6,97)}$= 1.1171, p= 0.3586**			
	$Elav^{C155}$-Gal4> w^{1118}	0.5216 ± 0.0383		
	$Elav^{C155}$-Gal4 >0N3R	0.5339 ± 0.0399	0.0363	0.8494
	$Elav^{C155}$-Gal4 >1N3R	0.5068 ± 0.0536	0.0574	0.8111
	$Elav^{C155}$-Gal4 >2N3R	0.4923 ± 0.0453	0.2349	0.6291
	$Elav^{C155}$-Gal4 >0N4R	0.4147 ± 0.0331	3.0037	0.0865
	$Elav^{C155}$-Gal4 >1N4R	0.4951 ± 0.0691	0.1684	0.6825
	$Elav^{C155}$-Gal4 >2N4R	0.4067 ± 0.0472	3.1687	0.0784

Supplemental Table S5.

The means and SEMs of habituation to footshock following exposure to 15-stimuli **(A)** or 2-stimuli **(B)** of the indicated genotypes are shown. ANOVA, followed by planned multiple comparisons using the least squares means (LSM) approach, are shown. Significant differences are highlighted in bold.

**Statistical details from Figure 5**				
	**Genotype**	**Mean ± SEM**	**F-Ratio**	**p**
**Figure 5A-15 stimuli**				
**0N3R**	**ANOVA $F_{(2,30)}$=1.2289, p=0.3079**			
	w^{1118}>0N3R	6.759 ± 0.474		
	$Elav^{C155}$-Gal4> w^{1118}	8.585 ± 1.718	0.8417	0.3667
	$Elav^{C155}$-Gal4 >0N3R	5.592 ± 1.491	0.3950	0.5348
	$Elav^{C155}$-Gal4> w^{1118}	8.585 ± 1.718		
	$Elav^{C155}$-Gal4 >0N3R	5.592 ± 1.491	2.4530	0.1285
**1N3R**	**ANOVA $F_{(2,26)}$= 4.2644, p=0.0260**			
	w^{1118}>1N3R	8.268 ± 2.053		
	$Elav^{C155}$-Gal4> w^{1118}	6.499 ± 0.794	0.4655	0.5016
	$Elav^{C155}$-Gal4 >1N3R	0.583 ± 2.686	7.8542	**0.0099**

	Elav^{C155}-Gal4> w^{1118}	6.499 ± 0.794		
	Elav^{C155}-Gal4 >1N3R	0.583 ± 2.686	4.8841	**0.0369**
**2N3R**	**ANOVA $F_{(2,45)}$= 2.4007, p=0.1027**			
	w^{1118}>2N3R	3.239 ± 1.427		
	Elav^{C155}-Gal4> w^{1118}	8.499 ± 1.972	4.6242	0.0372
	Elav^{C155}-Gal4 >2N3R	5.053 ± 1.734	0.5326	0.4695
	Elav^{C155}-Gal4> w^{1118}	8.499 ± 1.972		
	Elav^{C155}-Gal4 >2N3R	5.053 ± 1.734	1.9851	0.1660
**0N4R**	**ANOVA $F_{(2,29)}$=0.9303, p=0.4067**			
	w^{1118}>0N4R	5.481 ± 1.295		
	Elav^{C155}-Gal4> w^{1118}	8.585 ± 1.718	1.5410	0.2251
	Elav^{C155}-Gal4 >0N4R	8.151 ± 1.798	1.3025	0.2638
	Elav^{C155}-Gal4> w^{1118}	8.585 ± 1.718		
	Elav^{C155}-Gal4 >0N4R	8.151 ± 1.798	0.0345	0.8540
**1N4R**	**ANOVA $F_{(2,29)}$=1.5501, p=0.2305**			
	w^{1118}>1N4R	3.984 ± 1.145		
	Elav^{C155}-Gal4> w^{1118}	6.499 ± 0.794	1.1978	0.2834
	Elav^{C155}-Gal4 >1N4R	7.921 ± 2.183	3.0678	0.0912
	Elav^{C155}-Gal4> w^{1118}	6.499 ± 0.794		
	Elav^{C155}-Gal4 >1N4R	7.921 ± 2.183	0.4236	0.5206
**2N4R**	**ANOVA $F_{(2,45)}$=1.2392, p=0.2997**			
	w^{1118}>2N4R	5.061 ± 0.985		
	Elav^{C155}-Gal4> w^{1118}	8.499 ± 1.972	1.7249	0.1960
	Elav^{C155}-Gal4 >2N4R	4.847 ± 2.334	0.0065	0.9362
	Elav^{C155}-Gal4> w^{1118}	8.499 ± 1.972		
	Elav^{C155}-Gal4 >2N4R	4.847 ± 2.334	1.9465	0.1701
**Figure 5B-2 stimuli**				
**0N3R**	**ANOVA $F_{(2,29)}$=1.2393, p=0.3055**			
	w^{1118}>0N3R	-0.644 ± 1.503		
	Elav^{C155}-Gal4> w^{1118}	-3.631 ± 1.579	2.2204	0.1478
	Elav^{C155}-Gal4 >0N3R	-3.138 ± 1.121	1.3992	0.2472
	Elav^{C155}-Gal4> w^{1118}	-3.631 ± 1.579		
	Elav^{C155}-Gal4 >0N3R	-3.138 ± 1.121	0.0573	0.8127
**1N3R**	**ANOVA $F_{(2,28)}$=0.1282, p=0.8802**			
	w^{1118}>1N3R	-1.949 ± 1.613		
	Elav^{C155}-Gal4> w^{1118}	-3.498 ± 1.695	0.2561	0.6171

	$Elav^{C155}$-Gal4 >1N3R	-2.798 ± 2.721	0.0739	0.7879
	$Elav^{C155}$-Gal4> w^{1118}	-3.498 ± 1.695		
	$Elav^{C155}$-Gal4 >1N3R	-2.798 ± 2.721	0.0591	0.8098
**2N3R**	**ANOVA $F_{(2,25)}$= 13.7352, p=0.0001**			
	w^{1118}>2N3R	-2.212 ± 1.106		
	$Elav^{C155}$-Gal4> w^{1118}	-2.830 ± 2.226	0.0630	0.8040
	$Elav^{C155}$-Gal4 >2N3R	7.384 ± 1.489	18.853	**0.0002**
	$Elav^{C155}$-Gal4> w^{1118}	-2.830 ± 2.226		
	$Elav^{C155}$-Gal4 >2N3R	7.384 ± 1.489	19.727	**0.0002**
**0N4R**	**ANOVA $F_{(2,30)}$=9.2731, p=0.0008**			
	w^{1118}>0N4R	-2.013± 1.267		
	$Elav^{C155}$-Gal4> w^{1118}	-3.631± 1.579	0.3719	0.5469
	$Elav^{C155}$-Gal4 >0N4R	5.512± 1.792	8.5583	**0.0067**
	$Elav^{C155}$-Gal4> w^{1118}	-3.631± 1.579		
	$Elav^{C155}$-Gal4 >0N4R	5.512± 1.792	16.544	**0.0003**
**1N4R**	**ANOVA $F_{(2,33)}$=7.8347, p=0.0018**			
	w^{1118}>1N4R	-0.123± 0.708		
	$Elav^{C155}$-Gal4> w^{1118}	-3.498± 1.695	2.2555	0.1433
	$Elav^{C155}$-Gal4 >1N4R	5.491± 2.166	6.2410	**0.0180**
	$Elav^{C155}$-Gal4> w^{1118}	-3.498± 1.695		
	$Elav^{C155}$-Gal4 >1N4R	5.491± 2.166	15.333	**0.0005**
**2N4R**	**ANOVA $F_{(2,25)}$=0.5918, p=0.5615**			
	w^{1118}>2N4R	-0.565± 1.165		
	$Elav^{C155}$-Gal4> w^{1118}	-2.830± 2.226	0.8661	0.3617
	$Elav^{C155}$-Gal4 >2N4R	-0.351 ± 1.789	0.0089	0.9254
	$Elav^{C155}$-Gal4> w^{1118}	-2.830± 2.226		
	$Elav^{C155}$-Gal4 >2N4R	-0.351 ± 1.789	0.9927	0.3295

Supplemental Table S6.

The means and SEMs of the four 6-hour intervals per day (early day: 0600-1130, late day: 1200-1730, early night: 1800-2330 and late night 2400-0530 hours) of the indicated genotypes are shown. Following the indicated ANOVA, the means we compared using planned multiple comparisons. Significant differences are highlighted in bold.

Statistical details from Figure 6					
	Genotype		Mean ± SEM	F-Ratio	p
			**ANOVA $F_{(11,643)}$=43.3223,**		**p=6.1587e-70**
**0N3R**	0600-1130	w^{1118}>0N3R	138.08 ± 10.137		
		$Elav^{C155}$-Gal4> w^{1118}	144.96 ± 10.680	0.3217	0.5708
		$Elav^{C155}$-Gal4 >0N3R	199.47 ± 11.834	27.188	**2.50e-7**
		$Elav^{C155}$-Gal4> w^{1118}	144.96 ± 10.680		
		$Elav^{C155}$-Gal4 >0N3R	199.47 ± 11.834	21.891	**3.53e-6**
	1200-1730	w^{1118}>0N3R	48.79 ± 5.132		
		$Elav^{C155}$-Gal4> w^{1118}	47.69 ± 7.936	0.0082	0.9279
		$Elav^{C155}$-Gal4 >0N3R	61.78 ± 7.825	1.2171	0.2703
		$Elav^{C155}$-Gal4> w^{1118}	47.69 ± 7.936		
		$Elav^{C155}$-Gal4 >0N3R	61.78 ± 7.825	1.4619	0.2271
	1800-2330	w^{1118}>0N3R	95.51 ± 7.700		
		$Elav^{C155}$-Gal4> w^{1118}	101.23 ± 8.358	0.2223	0.6374
		$Elav^{C155}$-Gal4 >0N3R	131.90 ± 9.345	9.5509	**0.0021**
		$Elav^{C155}$-Gal4> w^{1118}	101.23 ± 8.358		
		$Elav^{C155}$-Gal4 >0N3R	131.90 ± 9.345	6.9281	**0.0087**
	2400-0530	w^{1118}>0N3R	37.94 ± 6.602		
		$Elav^{C155}$-Gal4> w^{1118}	38.80 ± 5.197	0.6461	0.4218
		$Elav^{C155}$-Gal4 >0N3R	31.76 ± 5.092	0.2752	0.6000
		$Elav^{C155}$-Gal4> w^{1118}	38.80 ± 5.197		
		$Elav^{C155}$-Gal4 >0N3R	31.76 ± 5.092	0.3646	0.5462
			**ANOVA $F_{(11,1151)}$=50.7056,**		**p=8.5036e-91**
**1N3R**	0600-1130	w^{1118}>1N3R	183.05 ± 11.940		
		$Elav^{C155}$-Gal4> w^{1118}	160.65 ± 8.206	3.2133	0.0733
		$Elav^{C155}$-Gal4 >1N3R	176.24 ± 7.693	0.2970	0.5858
		$Elav^{C155}$-Gal4> w^{1118}	160.65 ± 8.206		
		$Elav^{C155}$-Gal4 >1N3R	176.24 ± 7.693	1.5564	0.2124

	1200-1730	w^{1118}>1N3R	93.85 ± 10.996		
		$Elav^{C155}$-Gal4> w^{1118}	46.61 ± 8.111	14.283	0.0002
		$Elav^{C155}$-Gal4 >1N3R	78.68 ± 8.020	1.4733	0.2251
		$Elav^{C155}$-Gal4> w^{1118}	46.61 ± 8.111		
		$Elav^{C155}$-Gal4 >1N3R	78.68 ± 8.020	6.5818	**0.0104**
	1800-2330	w^{1118}>1N3R	156.64 ± 7.584		
		$Elav^{C155}$-Gal4> w^{1118}	154.62 ± 6.664	0.0260	0.8719
		$Elav^{C155}$-Gal4 >1N3R	238.41 ± 12.350	42.791	**9.18e-11**
		$Elav^{C155}$-Gal4> w^{1118}	154.62 ± 6.664		
		$Elav^{C155}$-Gal4 >1N3R	238.41 ± 12.350	44.926	**3.21e-11**
	2400-0530	w^{1118}>1N3R	86.09 ± 9.252		
		$Elav^{C155}$-Gal4> w^{1118}	41.02 ± 3.888	13.006	0.0003
		$Elav^{C155}$-Gal4 >1N3R	63.57 ± 7.884	3.2462	0.0718
		$Elav^{C155}$-Gal4> w^{1118}	41.02 ± 3.888		
		$Elav^{C155}$-Gal4 >1N3R	63.57 ± 7.884	3.2567	0.0714
			**ANOVA $F_{(11,979)}$=52.4175, p=1.709e-90**		
**2N3R**	0600-1130	w^{1118}>2N3R	181.17 ± 10.331		
		$Elav^{C155}$-Gal4> w^{1118}	157.76 ± 8.782	3.4645	0.0630
		$Elav^{C155}$-Gal4 >2N3R	195.15 ± 8.039	1.2423	0.2653
		$Elav^{C155}$-Gal4> w^{1118}	157.76 ± 8.782		
		$Elav^{C155}$-Gal4 >2N3R	195.15 ± 8.039	9.3575	**0.0023**
	1200-1730	w^{1118}>2N3R	106.60 ± 7.564		
		$Elav^{C155}$-Gal4> w^{1118}	58.79 ± 9.570	14.446	0.0001
		$Elav^{C155}$-Gal4 >2N3R	106 ± 9.370	0.0023	0.9619
		$Elav^{C155}$-Gal4> w^{1118}	58.79 ± 9.570		
		$Elav^{C155}$-Gal4 >2N3R	106 ± 9.370	14.915	**0.0001**
	1800-2330	w^{1118}>2N3R	171.57 ± 8.965		
		$Elav^{C155}$-Gal4> w^{1118}	144.76 ± 7.901	4.5399	0.0334
		$Elav^{C155}$-Gal4 >2N3R	233.46 ± 12.451	24.347	**9.47e-7**
		$Elav^{C155}$-Gal4> w^{1118}	144.76 ± 7.901		
		$Elav^{C155}$-Gal4 >2N3R	233.46 ± 12.451	52.645	**8.18e-13**
	2400-0530	w^{1118}>2N3R	62.67 ± 9.081		
		$Elav^{C155}$-Gal4> w^{1118}	51.89 ± 4.792	0.7341	0.3918
		$Elav^{C155}$-Gal4 >2N3R	46.21 ± 6.292	1.7215	0.1898
		$Elav^{C155}$-Gal4> w^{1118}	51.89 ± 4.792		

		$Elav^{C155}$-Gal4 >2N3R	46.21 ± 6.292	0.2159	0.6422
			**ANOVA $F_{(11,1139)}$=197.3919, p=9.27e-254**		
**0N4R**	0600-1130	w^{1118}>0N4R	271.16 ± 10.591		
		$Elav^{C155}$-Gal4> w^{1118}	260.46 ± 9.009	0.9076	0.3409
		$Elav^{C155}$-Gal4 >0N4R	348.52 ± 13.366	47.188	**1.06e-11**
		$Elav^{C155}$-Gal4> w^{1118}	260.46 ± 9.009		
		$Elav^{C155}$-Gal4 >0N4R	348.52 ± 13.366	61.793	**8.86e-15**
	1200-1730	w^{1118}>0N4R	61.96 ± 3.931		
		$Elav^{C155}$-Gal4> w^{1118}	46.48 ± 3.728	1.9001	0.1683
		$Elav^{C155}$-Gal4 >0N4R	44.47 ± 3.926	2.4131	0.1206
		$Elav^{C155}$-Gal4> w^{1118}	46.48 ± 3.728		
		$Elav^{C155}$-Gal4 >0N4R	44.47 ± 3.926	0.0322	0.8576
	1800-2330	w^{1118}>0N4R	190.38 ± 6.876		
		$Elav^{C155}$-Gal4> w^{1118}	183.22 ± 8.444	0.4056	0.5243
		$Elav^{C155}$-Gal4 >0N4R	232.4 ± 11.473	13.923	**0.0002**
		$Elav^{C155}$-Gal4> w^{1118}	183.22 ± 8.444		
		$Elav^{C155}$-Gal4 >0N4R	232.4 ± 11.473	19.269	**1.24e-5**
	2400-0530	w^{1118}>0N4R	46.69 ± 3.3662		
		$Elav^{C155}$-Gal4> w^{1118}	39.31 ± 4.387	0.4315	0.5113
		$Elav^{C155}$-Gal4 >0N4R	57.1 ± 7.704	0.8542	0.3556
		$Elav^{C155}$-Gal4> w^{1118}	39.31 ± 4.387		
		$Elav^{C155}$-Gal4 >0N4R	57.1 ± 7.704	2.5211	0.1126
			**ANOVA $F_{(11,1143)}$=152.5621, p=1.47e-214**		
**1N4R**	0600-1130	w^{1118}>1N4R	266.79 ± 8.565		
		$Elav^{C155}$-Gal4> w^{1118}	260.46 ± 9.009	0.2567	0.6125
		$Elav^{C155}$-Gal4 >1N4R	325.53 ± 15.903	22.075	**2.94e-6**
		$Elav^{C155}$-Gal4> w^{1118}	260.46 ± 9.009		
		$Elav^{C155}$-Gal4 >1N4R	325.53 ± 15.903	27.381	**1.99e-7**
	1200-1730	w^{1118}>1N4R	98.06 ± 5.547		
		$Elav^{C155}$-Gal4> w^{1118}	46.48 ± 3.728	17.024	3.96e-5
		$Elav^{C155}$-Gal4 >1N4R	84.12 ± 6.651	1.2423	0.2653
		$Elav^{C155}$-Gal4> w^{1118}	46.48 ± 3.728		
		$Elav^{C155}$-Gal4 >1N4R	84.12 ± 6.651	9.1654	**0.0025**
		w^{1118}>1N4R	243.01 ± 8.977		

	1800-2330	Elav^{C155}-Gal4> w^{1118}	183.22 ± 8.444	22.873	1.96e-6
		Elav^{C155}-Gal4 >1N4R	292.96 ± 12.951	15.968	**6.86e-5**
		Elav^{C155}-Gal4> w^{1118}	183.22 ± 8.444		
		Elav^{C155}-Gal4 >1N4R	292.96 ± 12.951	77.883	**4.07e-18**
	2400-0530	w^{1118}>1N4R	61.78 ± 4.593		
		Elav^{C155}-Gal4> w^{1118}	39.31 ± 4.387	3.2292	0.0726
		Elav^{C155}-Gal4 >1N4R	60.72 ± 8.572	0.0071	0.9329
		Elav^{C155}-Gal4> w^{1118}	39.31 ± 4.387		
		Elav^{C155}-Gal4 >1N4R	60.72 ± 8.572	2.9649	0.0854
**ANOVA $F_{(11,1135)}$=180.9182, p=1.17e-239**					
**2N4R**	0600-1130	w^{1118}>2N4R	233.05 ± 9.582		
		Elav^{C155}-Gal4> w^{1118}	260.46 ± 9.009	7.3152	0.0069
		Elav^{C155}-Gal4 >2N4R	258.04 ± 10.432	6.0508	**0.0140**
		Elav^{C155}-Gal4> w^{1118}	260.46 ± 9.009		
		Elav^{C155}-Gal4 >2N4R	258.04 ± 10.432	0.0575	0.8106
	1200-1730	w^{1118}>2N4R	81.74 ± 4.548		
		Elav^{C155}-Gal4> w^{1118}	46.48 ± 3.728	12.112	0.0005
		Elav^{C155}-Gal4 >2N4R	61.58 ± 4.867	3.9375	**0.0475**
		Elav^{C155}-Gal4> w^{1118}	46.48 ± 3.728		
		Elav^{C155}-Gal4 >2N4R	61.58 ± 4.867	2.2464	0.1342
	1800-2330	w^{1118}>2N4R	197.74 ± 8.693		
		Elav^{C155}-Gal4> w^{1118}	183.22 ± 8.443	2.0515	0.1523
		Elav^{C155}-Gal4 >2N4R	245.73 ± 9.171	22.321	**2.60e-6**
		Elav^{C155}-Gal4> w^{1118}	183.22 ± 8.443		
		Elav^{C155}-Gal4 >2N4R	245.73 ± 9.171	38.469	**7.80e-10**
	2400-0530	w^{1118}>2N4R	49.55 ± 3.351		
		Elav^{C155}-Gal4> w^{1118}	39.31 ± 4.387	1.0216	0.3123
		Elav^{C155}-Gal4 >2N4R	32.15 ± 3.644	2.9360	0.0869
		Elav^{C155}-Gal4> w^{1118}	39.31 ± 4.387		
		Elav^{C155}-Gal4 >2N4R	32.15 ± 3.644	0.5055	0.4772

Supplemental Table S7.

Mean mortalities after 24, 48, 52, 58 and 75 hours of exposure to 5% H_2O_2 of the indicated genotypes were compared with that of elavGAL4>w[1118] control animals following the indicated significant ANOVA with planned multiple comparisons as indicated. Significant differences are highlighted in bold.

Statistical details from Figure 7				
	Genotype	Mean ± SEM	F-Ratio	p
		**ANOVA $F_{(6,524)}$=10,3976, p=6,76e-11**		
24 Hours	$Elav^{C155}$-Gal4> w^{1118}	0.667 ± 0.454		
	$Elav^{C155}$-Gal4 >0N3R	0.667 ± 0.454	1.4e-32	1
	$Elav^{C155}$-Gal4 >1N3R	0.667 ± 0.454	1.4e-32	1
	$Elav^{C155}$-Gal4 >2N3R	0.333 ± 0.333	0.1310	0.7182
	$Elav^{C155}$-Gal4 >0N4R	2.333 ± 0.959	3.2754	0.0734
	$Elav^{C155}$-Gal4 >1N4R	3.333 ± 1.054	8.3850	**0.0047**
	$Elav^{C155}$-Gal4 >2N4R	0.667 ± 0.454	0	1
	$Elav^{C155}$-Gal4 >0N3R	0.667 ± 0.454		
	$Elav^{C155}$-Gal4 >1N3R	0.667 ± 0.454	0	1
	$Elav^{C155}$-Gal4 >2N3R	0.333 ± 0.333	0.1310	0.7182
	$Elav^{C155}$-Gal4 >0N4R	2.333 ± 0.959	3.2754	0.0734
	$Elav^{C155}$-Gal4 >1N4R	3.333 ± 1.054	8.3850	**0.0047**
	$Elav^{C155}$-Gal4 >2N4R	0.667 ± 0.454	1.4e-32	1
	$Elav^{C155}$-Gal4 >1N3R	0.667 ± 0.454		
	$Elav^{C155}$-Gal4 >2N3R	0.333 ± 0.333	0.1310	0.7182
	$Elav^{C155}$-Gal4 >0N4R	2.333 ± 0.959	3.2754	0.0734
	$Elav^{C155}$-Gal4 >1N4R	3.333 ± 1.054	8.3850	**0.0047**
	$Elav^{C155}$-Gal4 >2N4R	0.667 ± 0.454	1.4e-32	1
	$Elav^{C155}$-Gal4 >2N3R	0.333 ± 0.333		
	$Elav^{C155}$-Gal4 >0N4R	2.333 ± 0.959	4.7166	**0.0323**
	$Elav^{C155}$-Gal4 >1N4R	3.333 ± 1.054	10.612	**0.0015**
	$Elav^{C155}$-Gal4 >2N4R	0.667 ± 0.454	0.1310	0.7182
	$Elav^{C155}$-Gal4 >0N4R	2.333 ± 0.959		
	$Elav^{C155}$-Gal4 >1N4R	3.333 ± 1.054	1.1791	0.2802
	$Elav^{C155}$-Gal4 >2N4R	0.667 ± 0.454	3.2754	0.0734
	$Elav^{C155}$-Gal4 >1N4R	3.333 ± 1.054		
	$Elav^{C155}$-Gal4 >2N4R	0.667 ± 0.454	8.3850	**0.0047**
48 Hours	$Elav^{C155}$-Gal4> w^{1118}	2 ± 0.816		
	$Elav^{C155}$-Gal4 >0N3R	4 ± 1.558	0.8344	0.3632
	$Elav^{C155}$-Gal4 >1N3R	4 ± 1.215	0.8344	0.3632
	$Elav^{C155}$-Gal4 >2N3R	5.333 ± 1.420	2.3179	0.1311

	Elav^{C155}-Gal4 >0N4R	11.333 ± 1.241	18.172	**4.65e-5**
	Elav^{C155}-Gal4 >1N4R	16.667 ± 2.051	44.874	**1.33e-9**
	Elav^{C155}-Gal4 >2N4R	13.333 ± 2.108	26.794	**1.20e-6**
	Elav^{C155}-Gal4 >0N3R	4 ± 1.558		
	Elav^{C155}-Gal4 >1N3R	4 ± 1.215	0	1
	Elav^{C155}-Gal4 >2N3R	5.333 ± 1.420	0.3709	0.5439
	Elav^{C155}-Gal4 >0N4R	11.333 ± 1.241	11.218	**0.0011**
	Elav^{C155}-Gal4 >1N4R	16.667 ± 2.051	33.470	**8.67e-8**
	Elav^{C155}-Gal4 >2N4R	13.333 ± 2.108	18.172	**4.64e-5**
	Elav^{C155}-Gal4 >1N3R	4 ± 1.215		
	Elav^{C155}-Gal4 >2N3R	5.333 ± 1.420	0.3709	0.5439
	Elav^{C155}-Gal4 >0N4R	11.333 ± 1.241	11.218	**0.0011**
	Elav^{C155}-Gal4 >1N4R	16.667 ± 2.051	33.470	**8.66e-8**
	Elav^{C155}-Gal4 >2N4R	13.333 ± 2.108	18.172	**4.64e-5**
	Elav^{C155}-Gal4 >2N3R	5.333 ± 1.420		
	Elav^{C155}-Gal4 >0N4R	11.333 ± 1.241	7.5099	**0.0073**
	Elav^{C155}-Gal4 >1N4R	16.667 ± 2.051	26.795	**1.20e-6**
	Elav^{C155}-Gal4 >2N4R	13.333 ± 2.108	13.350	**0.0004**
	Elav^{C155}-Gal4 >0N4R	11.333 ± 1.241		
	Elav^{C155}-Gal4 >1N4R	16.667 ± 2.051	5.9338	**0.0167**
	Elav^{C155}-Gal4 >2N4R	13.333 ± 2.108	0.8344	0.3632
	Elav^{C155}-Gal4 >1N4R	16.667 ± 2.051		
	Elav^{C155}-Gal4 >2N4R	13.333 ± 2.108	2.3179	0.1311
**52 Hours**	Elav^{C155}-Gal4> w^{1118}	2.667 ± 0.826		
	Elav^{C155}-Gal4 >0N3R	5.333 ± 1.791	1.0228	0.3143
	Elav^{C155}-Gal4 >1N3R	7.333 ± 1.453	3.1324	0.0799
	Elav^{C155}-Gal4 >2N3R	8.333 ± 1.992	4.6187	**0.0341**
	Elav^{C155}-Gal4 >0N4R	15 ± 1.618	21.879	**9.30e-6**
	Elav^{C155}-Gal4 >1N4R	20.333 ± 2.153	44.893	**1.32e-9**
	Elav^{C155}-Gal4 >2N4R	15.667 ± 2.667	24.308	**3.35e-6**
	Elav^{C155}-Gal4 >0N3R	5.333 ± 1.791		
	Elav^{C155}-Gal4 >1N3R	7.333 ± 1.453	0.5753	0.4500
	Elav^{C155}-Gal4 >2N3R	8.333 ± 1.992	1.2945	0.2580
	Elav^{C155}-Gal4 >0N4R	15 ± 1.618	13.441	**0.0004**
	Elav^{C155}-Gal4 >1N4R	20.333 ± 2.153	32.363	**1.33e-7**
	Elav^{C155}-Gal4 >2N4R	15.667 ± 2.667	15.358	**0.0002**
	Elav^{C155}-Gal4 >1N3R	7.333 ± 1.453		
	Elav^{C155}-Gal4 >2N3R	8.333 ± 1.992	0.1438	0.7053
	Elav^{C155}-Gal4 >0N4R	15 ± 1.618	8.4543	**0.0045**
	Elav^{C155}-Gal4 >1N4R	20.333 ± 2.153	24.308	**3.35e-6**

	$Elav^{C155}$-Gal4 >2N4R	15.667 ± 2.667	9.9886	**0.0021**
	$Elav^{C155}$-Gal4 >2N3R	8.333 ± 1.992		
	$Elav^{C155}$-Gal4 >0N4R	15 ± 1.618	6.3927	**0.0131**
	$Elav^{C155}$-Gal4 >1N4R	20.333 ± 2.153	20.712	**1.53e-5**
	$Elav^{C155}$-Gal4 >2N4R	15.667 ± 2.667	7.7352	**0.0065**
	$Elav^{C155}$-Gal4 >0N4R	15 ± 1.618		
	$Elav^{C155}$-Gal4 >1N4R	20.333 ± 2.153	4.0913	**0.0458**
	$Elav^{C155}$-Gal4 >2N4R	15.667 ± 2.667	0.0639	0.8009
	$Elav^{C155}$-Gal4 >1N4R	20.333 ± 2.153		
	$Elav^{C155}$-Gal4 >2N4R	15.667 ± 2.667	3.1324	0.0799
**58 Hours**	$Elav^{C155}$-Gal4> w^{1118}	3.667 ± 1.031		
	$Elav^{C155}$-Gal4 >0N3R	8 ± 1.746	1.3728	0.2442
	$Elav^{C155}$-Gal4 >1N3R	12.333 ± 2.062	5.4914	**0.0211**
	$Elav^{C155}$-Gal4 >2N3R	14 ± 3.207	7.8065	**0.0063**
	$Elav^{C155}$-Gal4 >0N4R	21.333 ± 2.557	22.818	**6.25e-6**
	$Elav^{C155}$-Gal4 >1N4R	28.667 ± 2.947	45.694	**1.002e-9**
	$Elav^{C155}$-Gal4 >2N4R	22 ± 3.742	24.573	**2.999e-6**
	$Elav^{C155}$-Gal4 >0N3R	8 ± 1.746		
	$Elav^{C155}$-Gal4 >1N3R	12.333 ± 2.062	1.3728	0.2442
	$Elav^{C155}$-Gal4 >2N3R	14 ± 3.207	2.6320	0.1079
	$Elav^{C155}$-Gal4 >0N4R	21.333 ± 2.557	12.997	**0.0005**
	$Elav^{C155}$-Gal4 >1N4R	28.667 ± 2.947	31.226	**2.06e-7**
	$Elav^{C155}$-Gal4 >2N4R	22 ± 3.742	14.329	**0.0003**
	$Elav^{C155}$-Gal4 >1N3R	12.333 ± 2.062		
	$Elav^{C155}$-Gal4 >2N3R	14 ± 3.207	0.2031	0.6532
	$Elav^{C155}$-Gal4 >0N4R	21.333 ± 2.557	5.9219	**0.0168**
	$Elav^{C155}$-Gal4 >1N4R	28.667 ± 2.947	19.504	**2.59e-5**
	$Elav^{C155}$-Gal4 >2N4R	22 ± 3.742	6.8317	**0.0103**
	$Elav^{C155}$-Gal4 >2N3R	14 ± 3.207		
	$Elav^{C155}$-Gal4 >0N4R	21.333 ± 2.557	3.9317	**0.0502**
	$Elav^{C155}$-Gal4 >1N4R	28.667 ± 2.947	15.727	**0.0001**
	$Elav^{C155}$-Gal4 >2N4R	22 ± 3.742	4.6790	**0.0330**
	$Elav^{C155}$-Gal4 >0N4R	21.333 ± 2.557		
	$Elav^{C155}$-Gal4 >1N4R	28.667 ± 2.947	3.9317	0.0502
	$Elav^{C155}$-Gal4 >2N4R	22 ± 3.742	0.0325	0.8573
	$Elav^{C155}$-Gal4 >1N4R	28.667 ± 2.947		
	$Elav^{C155}$-Gal4 >2N4R	22 ± 3.742	3.2493	0.0745
	$Elav^{C155}$-Gal4> w^{1118}	18.667 ± 3.856		

75 Hours	Elav^{C155}-Gal4 >0N3R	35.333 ± 2.207	10.337	**0.0018**
	Elav^{C155}-Gal4 >1N3R	53 ± 4.163	43.868	**1.90e-9**
	Elav^{C155}-Gal4 >2N3R	49.667 ± 4.792	35.764	**3.63e-8**
	Elav^{C155}-Gal4 >0N4R	57.333 ± 3.712	55.641	**3.54e-11**
	Elav^{C155}-Gal4 >1N4R	58.667 ± 2.823	59.544	**1.02e-11**
	Elav^{C155}-Gal4 >2N4R	68.667 ± 3.501	93.038	**7.11e-16**
	Elav^{C155}-Gal4 >0N3R	35.333 ± 2.207		
	Elav^{C155}-Gal4 >1N3R	53 ± 4.163	11.615	**0.0009**
	Elav^{C155}-Gal4 >2N3R	49.667 ± 4.792	7.6456	**0.0068**
	Elav^{C155}-Gal4 >0N4R	57.333 ± 3.712	18.012	**4.99e-5**
	Elav^{C155}-Gal4 >1N4R	58.667 ± 2.823	20.262	**1.86e-5**
	Elav^{C155}-Gal4 >2N4R	68.667 ± 3.501	41.350	**4.66e-9**
	Elav^{C155}-Gal4 >1N3R	53 ± 4.163		
	Elav^{C155}-Gal4 >2N3R	49.667 ± 4.792	0.4135	0.5217
	Elav^{C155}-Gal4 >0N4R	57.333 ± 3.712	0.6988	0.4052
	Elav^{C155}-Gal4 >1N4R	58.667 ± 2.823	1.1950	0.2770
	Elav^{C155}-Gal4 >2N4R	68.667 ± 3.501	9.1343	**0.0032**
	Elav^{C155}-Gal4 >2N3R	49.667 ± 4.792		
	Elav^{C155}-Gal4 >0N4R	57.333 ± 3.712	2.1874	0.1423
	Elav^{C155}-Gal4 >1N4R	58.667 ± 2.823	3.0144	0.0857
	Elav^{C155}-Gal4 >2N4R	68.667 ± 3.501	13.435	**0.0004**
	Elav^{C155}-Gal4 >0N4R	57.333 ± 3.712		
	Elav^{C155}-Gal4 >1N4R	58.667 ± 2.823	0.0662	0.7975
	Elav^{C155}-Gal4 >2N4R	68.667 ± 3.501	4.7801	**0.0312**
	Elav^{C155}-Gal4 >1N4R	58.667 ± 2.823		
	Elav^{C155}-Gal4 >2N4R	68.667 ± 3.501	3.7215	0.0566

Supplemental Table S8.

Log-rank tests were used to compare survival curves of the indicated genotypes with that of elav^{c155}-GAL4 heterozygotes (w^{1118}) as indicated. Significant differences are highlighted in bold.

**Statistical details from Figure 8**						
	**Log-Rank comparison Elav^{C155}-Gal4>w^{1118} vs Elav^{C155}-Gal4>TAU**					
**Genotype / DAY**	**0N3R**	**1N3R**	**2N3R**	**0N4R**	**1N4R**	**2N4R**
1	0	0	0	0	0	0
3	0.0726	0.2878	0.753	0.9483	0.0637	0.2878
5	0.4067	0.1996	0.5115	0.9929	0.169	0.1753
7	0.1797	**0.0224**	0.5792	0.2357	0.9052	0.117
9	0.2614	**0.0224**	0.5792	0.2357	0.4612	0.117
11	0.2614	**0.0224**	0.2034	0.2357	0.4612	0.117
13	0.2888	**0.0153**	0.3941	0.1526	0.5657	0.099
15	0.1131	**0.0045**	0.2957	0.0628	0.3228	0.016
17	0.0862	**0.0306**	0.3215	0.1376	0.3691	0.0247
19	0.0862	**0.0306**	0.164	0.1829	0.3561	0.0582
21	0.0134	**0.0134**	0.4224	0.0765	0.7528	0.0229
23	0.5548	0.1904	0.2785	0.4116	0.1821	0.4713
25	**<0.0001**	0.7236	0.1838	**0.0121**	0.0952	**<0.0001**
27	**<0.0001**	**0.0003**	**<0.0001**	**<0.0001**	**0.0018**	**<0.0001**
29	**0.001**	0.0846	**0.0024**	**0.0013**	0.0638	**0.0003**
31	**0.007**	0.1054	**0.0027**	**0.0002**	0.2564	**0.0027**
33	0.0726	0.0726	0.3974	0.0825	0.4534	0.0726
35	0	0	0	0	0	0
